# Long-term survival after multidisciplinary treatments for HER2-positive advanced gastric cancer with multiple liver and lung metastases

**DOI:** 10.1186/s40792-023-01714-8

**Published:** 2023-08-07

**Authors:** Satoshi Yoshioka, Naoto Takahashi, Muneharu Fujisaki, Kenji Takeshita, Yuta Takano, Fumiaki Yano, Naoki Toya, Ken Eto

**Affiliations:** 1 Department of Surgery, The Jikei University Kashiwa Hospital, 163-1 Kashiwashita, Kashiwa, Chiba 277-8567 Japan; 2 Department of Surgery, The Jikei University School of Medicine, 3-19-18 Nishi-Shinbashi, Minato-Ku, Tokyo, 105-8471 Japan

**Keywords:** HER2-positive gastric cancer, Multiple metastases, Trastuzumab

## Abstract

**Background:**

Trastuzumab-based chemotherapy is a standard treatment regimen for human epithelial growth factor 2 (HER2)-positive gastric cancer. This is a case of a patient who has survived 12 years after being diagnosed with advanced gastric cancer with multiple liver and lung metastases.

**Case presentation:**

A woman in her 70s underwent total gastrectomy, cholecystectomy, and left hepatic lobectomy for gastric cancer with liver metastasis. One month after the surgery, multiple liver metastases appeared. After two courses of S-1 + CDDP chemotherapy, the liver metastases disappeared, and new lung metastases occurred. Because the primary tumor was HER2 positive, S-1 + CDDP + trastuzumab chemotherapy was performed. After one course of chemotherapy, the blood test showed pancytopenia, and CDDP was discontinued. S-1 + trastuzumab chemotherapy was then initiated, and as a result, the lung metastases disappeared. The patient is alive without recurrence 12 years after the surgery.

**Conclusions:**

We encountered a case of long-term survival after multidisciplinary treatments for HER2-positive advanced gastric cancer with multiple liver and lung metastases.

## Background

HER2-positive gastric cancer is found in about 15–20% of all gastric cancer cases. It has been reported that the HER2-positive status is an independent poor prognostic factor in gastric cancer patients who underwent surgical resection [1]. Since the publication of the ToGA trial, trastuzumab-based chemotherapy has been considered a standard treatment for HER2-positive advanced gastric cancer [2]. We report a case of 12-year survival after multidisciplinary treatments for HER2-positive advanced gastric cancer with multiple liver and lung metastases.

## Case presentation

**Fig. 1:**
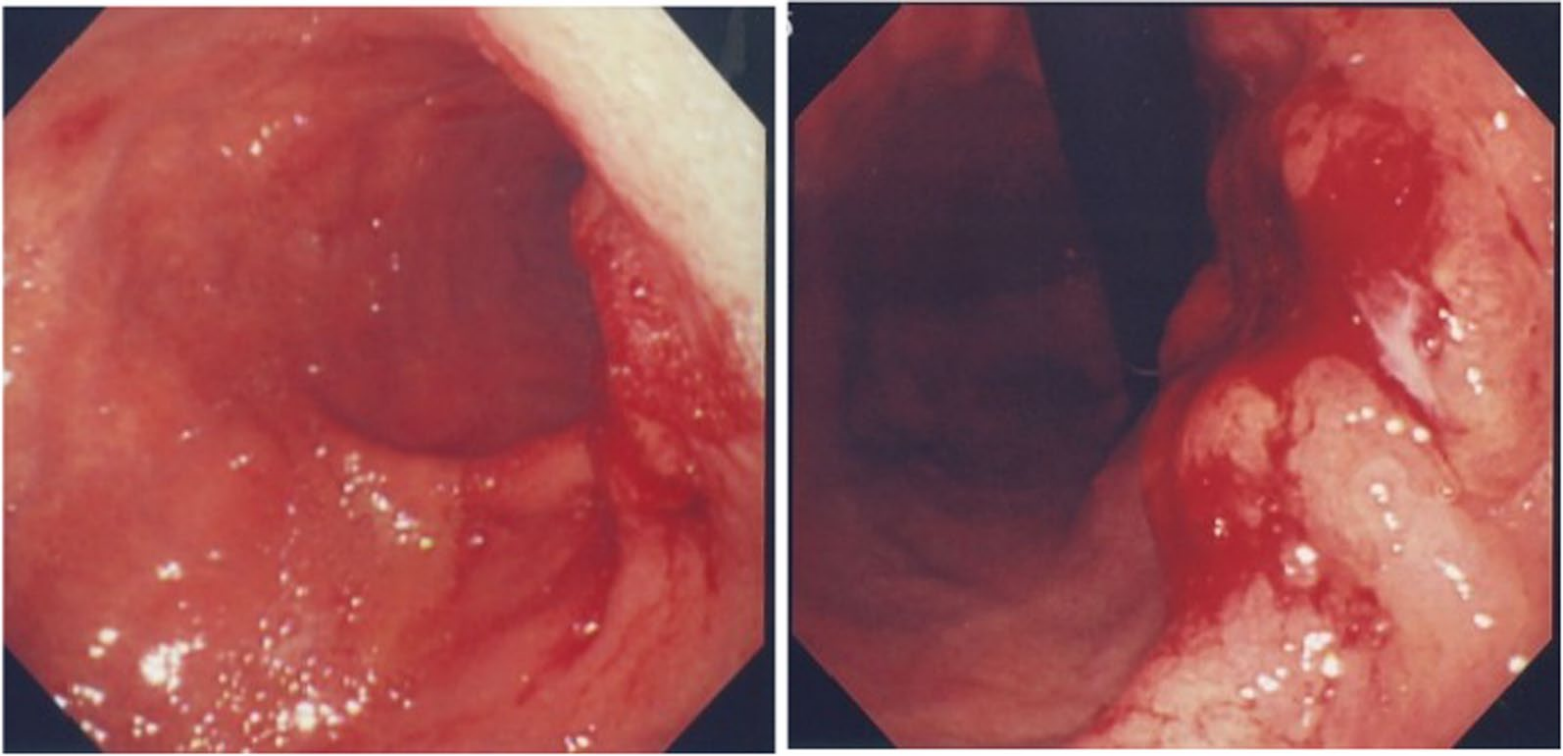
Upper gastrointestinal endoscopy. There was a type 2 tumor in the posterior wall of the upper body of the stomach

**Fig. 2:**
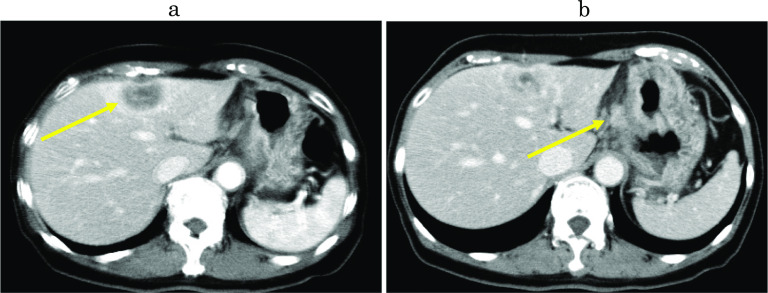
Preoperative abdominal CT imaging. **a** Enhanced CT showed an irregular enhanced nodule in the left lobe of the liver (yellow arrow). **b** The enlarged lymph nodes along the lesser curvature of the stomach were detected (yellow arrow)

**Fig. 3:**
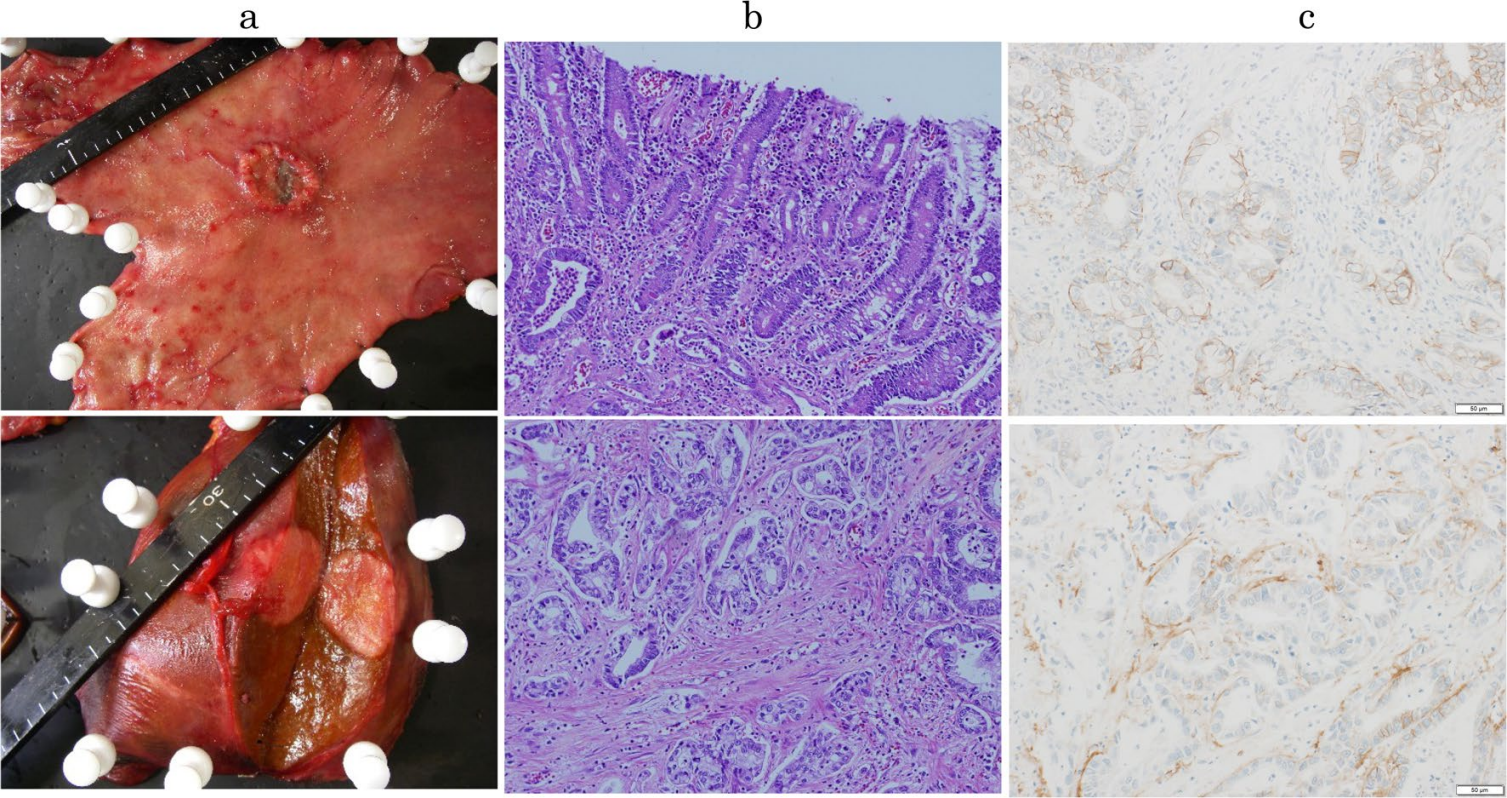
Pathological findings. **a** Resected specimens showed Type 2 advanced gastric cancer and liver metastasis in the left hepatic lobe. **b** Pathological findings showed tubular adenocarcinoma moderately differentiated. **c** Immunohistochemical staining showed that HER2 status was 2 +

**Fig. 4:**
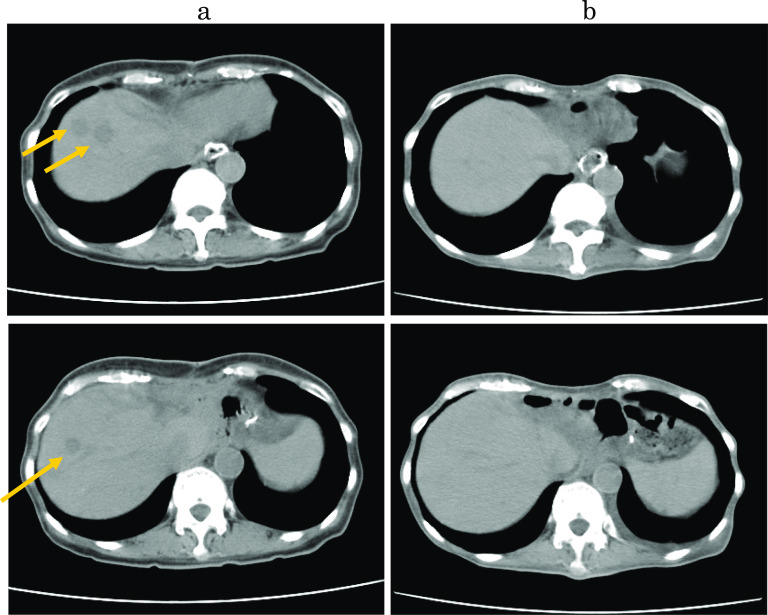
Abdominal CT imaging. **a** CT scan showed multiple liver metastases in the right lobe of the liver (yellow arrow). **b** The liver metastases disappeared after the S-1 plus CDDP chemotherapy

**Fig. 5:**
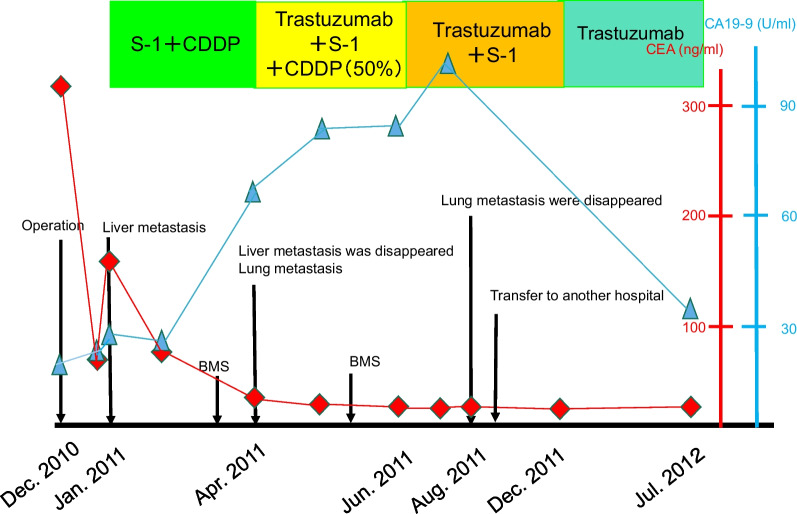
Diagram showing treatments and tumor markers. *CEA* carcinoembryonic antigen, *BMS* bone marrow suppression, *CDDP* cisplatin

A woman in her 70s visited our hospital with a complaint of hematemesis. Upper gastroscopy revealed a type 2 tumor in the posterior wall of the upper body of the stomach (Fig. 1). Biopsy revealed a moderately differentiated tubular adenocarcinoma. Computed tomography (CT) showed that the lymph nodes were enlarged along the lesser curvature of the stomach, with a metastatic lesion in the left lobe of the liver (Fig. 2a, b). The clinical diagnosis was gastric body adenocarcinoma with lymph node metastasis and liver metastasis. According to the Japanese classification of gastric carcinoma [3], the clinical stage was cT3N1H1P0, stage IV. The patient underwent total gastrectomy, D2 lymph node dissection, cholecystectomy, and left hepatic lobectomy (Fig. 3a) with R0 resection. Postoperative pathology was diagnosed as pT3N3aH1CY0P0, stage IV (Fig. 3b). However, one month after the surgery, multiple liver metastases appeared (Fig. 4a) and CEA has risen again (Fig. 5). After two courses of S-1 + CDDP chemotherapy (S-1 80 mg day1-21, CDDP 60 mg/m^2^ day 8), the liver metastases disappeared (Fig. 4b) [4] and CEA has dropped within the normal range (Fig. 5), but new lung metastases appeared (Fig. 4c). Because the primary tumor was HER2 positive (immunohistochemistry (IHC) 2 + /fluorescence in situ hybridization-positive), S-1 + CDDP + trastuzumab chemotherapy (S-1 80 mg day 1–21, CDDP 30 mg/m^2^ day 8, trastuzumab 174 mg) [5] was performed. Since S-1 Plus CDDP therapy was effective for liver metastasis, so we did not dare to change from S1 plus CDDP to capecitabine plus CDDP. After one course of chemotherapy, the blood test showed pancytopenia, and CDDP was discontinued. The lung metastasis disappeared after four courses of S-1 + trastuzumab chemotherapy (Fig. 4d). Considering bone marrow function and general status, S-1 + trastuzumab chemotherapy was continued until 1 year postoperatively, followed by trastuzumab alone for 1 year before chemotherapy was completed. The patient is alive without recurrence 12 years after the surgery.

## Discussion

Hepatic metastases are diagnosed synchronously in 3–14% of patients with gastric cancer [6]. Liver metastasis from gastric cancer (LMGC) is a systemic disease with an unfavorable prognosis. Systemic chemotherapy is considered the first therapeutic option for LMGC, but it fails to achieve satisfactory outcomes. The Japan Clinical Oncology Group reported a 5-year OS rate of only 1.7% in patients with metastasis confined to the liver and treated with systemic chemotherapy alone [7, 8]. On the other hand, there are highly selected cases when such metastases are found to be clinically resectable. Markar et al. reported that the median 1-year, 3-year, and 5-year survival were 68%, 31%, and 27%, respectively [6]. Guner et al. [9] suggested that the indications for liver resection are the following: no extrahepatic hematogenous metastasis; no peritoneal dissemination; the possibility of complete eradication of liver metastasis after liver resection; and adequate primary tumor control with complete removal of the primary gastric tumor and lymph nodes for synchronous metastasis. Since these indications were met in this case, total gastrectomy with D2 lymph node resection plus hepatectomy was carried out.

Surgery has potential benefits for a subset of patients with hepatic metastases, but hepatectomy for gastric cancer is still debated. This is because the high hepatic recurrence rate of 15–94% and general recurrence rate of 55–96% of LMGC imply that micrometastases remain in situ after surgery [10].

**Fig. 6:**
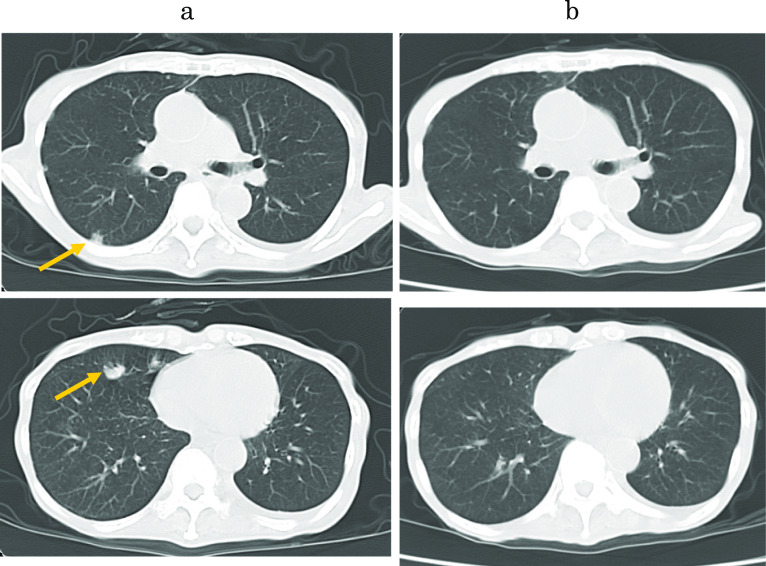
Thoracic CT imaging. **a** CT scan showed multiple lung metastases in the right lung (yellow arrow). **b** The lung metastases disappeared after S-1 plus trastuzumab chemotherapy

There are only limited data regarding pulmonary metastasis from gastric cancer. Kong et al. [11] reported an overall rate of pulmonary metastases of 0.96% (*n* = 20,187). They found that pulmonary metastases were most commonly the result of hematogenous metastasis which was significantly associated with liver metastases. In this case, we diagnosed the lung metastasis because nodular shadows appeared on chest CT after liver metastasis occurred in the early postoperative period and CA19-9 was also elevated (Fig. 5). But attention was required because diagnostic imaging was different from the typical lung metastasis of gastric cancer. Typical radiological findings of lung metastases include multiple rounds, variable-sized nodules and diffuse thickening of the stroma. However, atypical radiological features of metastases are often encountered, making it difficult to distinguish metastases from other non-malignant lung diseases [12]. In this case, the radiological characteristics of the lung tumor (Fig. 6) mimicked pneumonia, making it difficult to differentiate it from infection. Seo [12] and Abe [13] reported that the radiological findings of metastases from an adenocarcinoma of the gastrointestinal tract indicate air-space nodules, consolidation containing an air bronchogram, and focal or extensive ground-glass opacities.

In this case, hepatic recurrence and lung metastasis that occurred in the early postoperative period were severe problems. However, S-1 + CDDP chemotherapy followed by trastuzumab combination chemotherapy was successful, resulting in CR, and long-term survival without chemotherapy was obtained. The pathological findings of both the primary lesion and liver metastasis were HER2-positive, and trastuzumab was effective against lung metastasis, suggesting that lung metastasis also had HER2-positive status. Bozzetti et al. supported our speculation that the HER2 status of the primary tumor of gastric cancer and corresponding metastases are maintained and unchanged during the metastatic process [14]. The overall survival time for HER2-positive unresectable gastric cancer was 16.0 months in the ToGA trial [2], which was better than the 11.8 months for negative cases, but it was even longer at 12 years in this case. In the ToGA trial, a CR rate of 5% has been reported; however, there have not been many studies on the complete response in HER2-positive patients with gastric cancer with Herceptin. There is not enough data on the outcome of treatment after a complete response, so it cannot be said definitively. Even if a complete response is achieved, it is important to select an appropriate treatment based on factors such as the patient’s condition and risk of recurrence. This case is considered an exceptional long-term survivor of Her2-positive Stage IV advanced gastric cancer. The long-term survival is because there was no peritoneal metastasis and no recurrence in the residual liver and lungs after CR. In this case, a possible explanation for achieving CR is that the HER-2-positive status did not change during chemotherapy [15, 16].

Under the current guidelines, there is an option that the expression of HER2 should have been examined before surgery, and conversion surgery should have been attempted. In other words, gastrectomy plus hepatectomy without chemotherapy should have been avoided. However, this case underwent surgery in 2010 before Herceptin (Roche, Basel Switzerland) was approved in Japan in March 2011. There is no comprehensive report of conversion surgery for LMGC, and prospective studies with appropriate eligibility criteria are required. Several studies of conversion surgery for LMGC have recently been reported [8, 10, 17]. Fujitani et al. [8] supposed that tumor progression on preoperative chemotherapy was demonstrated to be associated with a poor outcome, even after potentially curative hepatectomy, suggesting that tumor control before surgery was crucial to offer a chance of prolonged remission. The response to neoadjuvant chemotherapy should be considered an unfavorable prognostic index, thus avoiding additional surgery [17, 18]. In the future, perioperative chemotherapy using trastuzumab, trastuzumab deruxtecan [19] as an antibody–drug conjugate, ramucirumab as an angiogenesis inhibitor, or anti-PD-1 antibody [20, 21] and anti-PDL-1 antibody as an immune check inhibitor is expected to enhance the antitumor effect and improve surgical outcomes.

## Conclusion

We report a case of long-term survival after multidisciplinary treatments for HER2-positive advanced gastric cancer with multiple liver and lung metastases.

## Data Availability

All data generated or analyzed during this study are included in this published article.

## Abbreviations

HER2: Human epithelial growth factor 2
CDDP: Cisplatin
CEA: Carcinoembryonic antigen
BMS: Bone marrow suppression
